# The Effects of a Multi-Component School-Based Nutrition Education Intervention on Children’s Determinants of Fruit and Vegetable Intake

**DOI:** 10.3390/nu14204259

**Published:** 2022-10-12

**Authors:** Marla T. H. Hahnraths, Jorieke P. M. Jansen, Bjorn Winkens, Onno C. P. van Schayck

**Affiliations:** 1 Department of Family Medicine, Care and Public Health Research Institute (CAPHRI), Maastricht University, P.O. Box 616, 6200 MD Maastricht, The Netherlands; 2 Department of Methodology and Statistics, Care and Public Health Research Institute (CAPHRI), Maastricht University, P.O. Box 616, 6200 MD Maastricht, The Netherlands

**Keywords:** primary school, health promoting school, nutrition, eating habits

## Abstract

Evidence suggests that multi-component school-based health-promoting interventions have great potential to improve children’s fruit and vegetable intake. However, interventions that combine classroom-based curricula with experiential learning strategies (e.g., cooking) are relatively seldom described. This study investigates the short-term and longer-term effects of a multi-component school-based nutrition education intervention combining classroom-based and experiential learning strategies on children’s determinants of their fruit and vegetable intake (knowledge, taste preferences, attitudes, and intention). Using a comparative quasi-experimental study design, data were collected, via child-reported questionnaires, at the baseline, directly after the intervention, and three months after the intervention from 4 control and 15 intervention classes from Dutch primary schools. A total of 192 children in grades three and four (aged 8–10 years) constituted the participants. After correction for the baseline, sex, age, and the fruit or vegetable product assessed in the questionnaire; the intervention group showed a significant increase in knowledge (*p* = 0.001; standardized effect size (ES = 0.60), taste preference (*p* = 0.002; ES = 0.52), attitude towards the assessed fruit or vegetable product (*p* = 0.004; ES = 0.48), and general attitude towards healthy products (*p* = 0.01; ES = 0.39) over the short term, when compared to the control group. The effects of the intervention did not continue to be significant over the longer term. The findings implicate short-term intervention success, although more research and intervention adaptations are recommended to increase the impact of such programs, especially over the long term.

## 1. Introduction

Fruit and vegetables (FVs) are crucial parts of a healthy eating pattern, and insufficient FV intake is related to a wide variety of health problems such as obesity, hypertension, cancer, and coronary heart disease [1,2]. Lifestyle behaviors formed during childhood are often maintained throughout the life course and promoting sufficient and varied FV consumption from a young age is, therefore, likely to result in both short-term and long-term health benefits [3,4,5,6]. Nevertheless, Dutch children’s FV consumption habits are suboptimal. Between 2014 and 2016, roughly 40% of 4–9-year-olds consumed at least 150 g of fruit (42%) or vegetables (41%) per day. For 9–12-year-olds, these percentages dropped even further to 20% for fruit and 25% for vegetables [7] per day. These numbers show significant room for improvement, underpinning the importance of efforts to promote children’s FV consumption.

There are various factors influencing children’s FV intake, and according to social cognitive theory (SCT) they can be categorized into personal determinants and environmental determinants [8]. Important personal factors include children’s taste preference and knowledge (e.g., concerning intake recommendations or different types of FV). Additionally, FV-related attitudes and self-efficacy are found to be associated with children’s FV intake [9,10,11,12]. With regard to environmental determinants, the availability and accessibility of FVs are positively related to consumption [10]. Additionally, (parental) modelling and peer influences play an important role, and the nutrition-related habits and behaviors of parents and peers shape children’s (perceived) social norms related to FV consumption [9]. Furthermore, nutrition-related rules and practices in, for example, the home and school setting influence children’s intake.

The school is a key environment when it comes to shaping children’s eating patterns. As children from different backgrounds come together regularly at school for several of their critical developmental years under the guidance of teachers who can serve as role models, schools can play an important role in the promotion of children’s FV intake [13,14,15]. Over the years, various school-based interventions have been developed and implemented to increase children’s FV consumption. Research has shown that interventions using either traditional educational strategies (e.g., explaining the health benefits of FV or tasting food products in school settings) or experiential learning strategies (e.g., garden- or cooking-related activities) have moderately positive effects on the determinants of FV intake among children [16,17,18,19,20]. Although evidence suggests that multi-component school-based interventions have the greatest potential to improve children’s FV intake, interventions combining a classroom-based curriculum with experiential learning strategies (e.g., cooking) are still relatively seldom described [16,21,22].

The Dutch “Kokkerelli learning street” program, developed by Kids University for Cooking, is a school-based nutrition education program that combines traditional education and experiential learning. The curriculum is developed for children in study years 5–8 in primary school (8–12 years old) and aims to improve children’s FV-related knowledge, taste preferences, attitudes, and intention. It comprises nine different “learning streets”: separate three-week programs which each focus on one specific FV product. All learning streets include classroom-based lessons, a visit to a grower’s farm, and a cooking workshop. Although the program is already being implemented in the Netherlands, its effectiveness has not yet been evaluated. The aim of the present study is, therefore, to investigate the effects of children’s participation in a learning street program on the various behavioral determinants of FV consumption. More specifically, the study aims to answer the following research questions: In comparison with children who did not participate in a learning street, what are the short-term (directly after the learning street) and longer-term (three months after the learning street) effects of participation in a learning street program on children’s:

- FV-related knowledge;
- FV-related taste preferences;
- FV-related attitudes;
- Attitudes related to healthy dietary habits in general;
- Intention to consume FV.

## 2. Materials and Methods

### 2.1. Intervention

**Figure 1:**
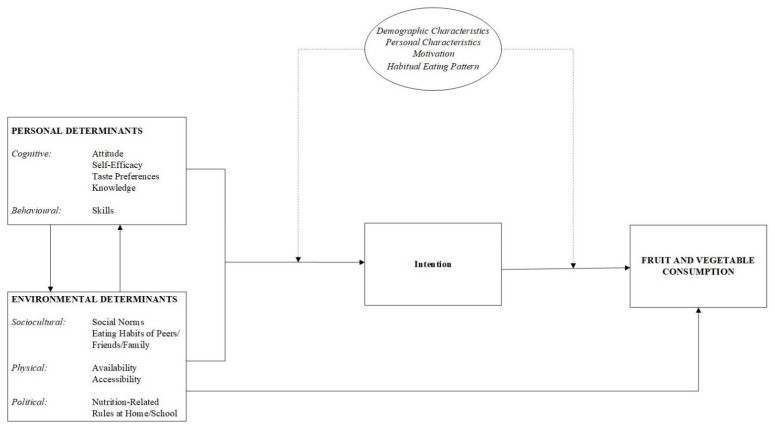
Conceptual framework of the determinants of children’s FV consumption (adapted from [8,23]).

Kids University for Cooking developed a school-based nutrition education curriculum specifically for children in study years 5–8 in primary school (internationally comparable to grades 3–6). A conceptual framework (Figure 1) based on SCT [8] and the environmental research framework for weight gain prevention (EnRG) [23] served as the theoretical foundation for the curriculum, which employs concepts from self-determination theory, experiential learning, and imagineering (e.g., self-experience in an interactive environment, rather than conventional education) [24,25,26]. The aim of the curriculum is to teach children where FVs come from, how they are processed, how they can be used for the preparation of healthy meals, and to let children experience different aspects of FVs in a positive context.

The curriculum consists of various so-called learning streets (nine in total), which all focus on one specific FV product: cauliflower, tomato, asparagus, pepper, strawberry, blueberry, mushroom, carrot, or leek. A school class can participate in a learning street as a one-time activity, but schools are encouraged to implement at least one learning street each study year to create a continuous curriculum for children from study years 5–8. Each learning street consists of multiple components, which schools can plan in a way that suits their schedule (although all activities should be planned in a specific order and within a three-week period). The program starts with an introduction lesson at school, during which the teacher introduces children to the taste of the specific product. Next, children visit a grower’s farm, where they receive information about the planting, growing, and harvesting processes. After visiting the grower’s farm, children prepare a dish containing the specific FV product at the cooking facilities of Kids University for Cooking with the help of a professional chef and volunteers. Afterward, they eat the self-prepared meal together with their peers. The last component is an evaluation lesson, which, again, takes place at school under the guidance of a teacher. A detailed overview of the different components of the program and its theoretical foundations are presented in Supplementary Tables S1 and S2.

### 2.2. Study Design

A quasi-experimental study was conducted. The need for ethical approval for the study has been waived by the FHML-REC in Maastricht, the Netherlands (FHML-REC/2019/062). The study was registered in the ClinicalTrials.gov database on 6 December 2019 (NCT04190680). The participants in the intervention group participated in a learning street, while the control group received no intervention and continued with their regular curriculum. The trial was non-randomized; primary school classes that registered to participate in a learning street for the first time during school years 2019–2020 or 2020–2021 were included in the intervention group. The school classes included in the control group were chosen from a pool of primary schools already participating in another research project on the effects of school-based health-promoting initiatives on children’s health and well-being (no publications on this study are available as the study is still ongoing). From this pool, classes from schools that were not (planning on) implementing nutrition-related and/or physical activity (PA)-related health-promoting initiatives in the context of this project were eligible to be included in the control group of the present study. Prior to the study’s start, an effect calculation was performed using the following assumptions: (a) children are nested within school classes, with 25 participants per class (as is common in the Netherlands), and seven intervention classes and seven control classes were included in the study; (b) an intraclass correlation coefficient of 0.04, a dropout rate of 10%, a standard deviation of 0.2, a power of 80%, and a significance level (alfa) of 0.05 were present in accordance with comparable research [27,28]. Based on these numbers, an effect size of 0.104 can be detected with sufficient power. This effect corresponds to a standardized effect size (Cohen’s d) of 0.52, indicating a medium effect size [29].

### 2.3. Participants and Recruitment

Participants were children in study years five and six (internationally comparable to grades three and four; aged 8–10 years) from primary schools located in Northern Limburg, the Netherlands. Researchers and employees of Kids University for Cooking informed the children and parents about the study both orally and via information brochures (containing information about the study’s purpose, procedures, and data handling). Informed consent was obtained from all parents of the participating children.

### 2.4. Data Collection Procedures

Data collection in the intervention group took place before the start of the introduction lesson (T0), directly after finishing the evaluation lesson (T1), and three months after the evaluation lesson (T2). For the control group, the same data collection timing was used, meaning there were three weeks between T0 and T1 data collection and three months between T1 and T2 data collection. Initially, data collection was to be completed in the school year 2019–2020, but due to the COVID-19 outbreak and the resulting closure of primary schools in the Netherlands, this was not feasible. Measurements in the intervention classes started before the COVID-19 outbreak in the school year 2019–2020 (September 2019). It was originally planned to include more intervention classes throughout the rest of the school year 2019–2020 and to also collect data in the control classes later during the school year 2019–2020. However, due to the mandatory primary school closure and the focus on minimizing the educational delay after reopening, data collection in both intervention and control classes was suspended from March 2020 until the end of the school year (July 2020). Measurements in the control classes were therefore conducted in the school year 2020–2021. However, as the learning streets were still suspended due to the ongoing COVID-19 outbreak, it was not possible to include more intervention classes during this school year. During all of the time points, children were asked to fill out the same short questionnaire in writing, assessing the various psychosocial determinants related to the FV product they encountered during their participation in the learning street. In the control group, participants received questionnaires concerning the same FV products that were addressed in the intervention group. For some of the classes from the control group, this was not possible, as COVID-19-related changes were made during planning. As a consequence of these changes, data collection in these classes was executed before it was known that no other intervention classes would be included in the study. Participants filled out the questionnaires during class hours, which took about 10 min. Due to the COVID-19-related restrictions, the participants filled out the questionnaires under the supervision of the responsible teacher only, who received written and verbal instructions from the researchers prior to data collection.

#### 2.4.1. Covariates

At baseline, participants’ demographic characteristics (age in years, sex (male/female), and (parental) birth country) were collected via the questionnaire. The children’s ethnicity was determined by parental birth country and categorized into (1) western background (i.e., the Netherlands and all other European countries (excluding Turkey), and North America, Japan, Indonesia, and Oceania) and (2) non-western background [30]. If at least one of the parents was born in a non-western country, the child’s ethnicity was assigned to non-western.

#### 2.4.2. Outcomes

Changes in the various psychosocial determinants of FV intake were selected as outcome measures. Based on the conceptual framework used to develop the learning street curriculum, five relevant determinants were selected: (i) knowledge; (ii) taste preferences; (iii) intention; (iv) attitude towards the FV item addressed in the learning street; and (v) attitude towards healthy food products in general [9,10,23]. Mean scores per participant on the various determinants of FV intake at each time point were calculated. At least two-thirds (67%) of the questions concerning a determinant had to be answered before a mean score for that determinant was calculated. The children’s knowledge was assessed by six true/false questions based on the information provided in the learning street. A correct answer was scored as 1; an incorrect answer was scored as 0. A mean summary score of the number of correct answers was computed by dividing the number of correct answers by the total number of items that were answered (mean summary score could range from 0 (low knowledge) to 6 (high knowledge)). Three questions were developed regarding taste preferences (e.g., “What do you think about the taste of the FV product?”) (five-point Likert scale from 1 = “I do not like it” to 5 = “I like it very much”, with an additional answer option “never tried”). Mean taste preferences were calculated by adding the scores of the questions that were answered and dividing them by the amount of answered questions (mean summary score could range from 1 (low taste preferences) to 5 (high taste preferences)). Two questions were used to assess intention, concerning participants’ plans to consume or cook a meal containing the FV product, and were assessed on a five-point Likert scale from 1 = “no, I do not want to” to 5 = “yes I want to” with an additional answer option “I do not know”. Mean intention was calculated by adding the scores of the questions that were answered and dividing them by the amount of answered questions (mean summary score could range from 1 (low intention) to 5 (high intention)). The two questions and scales for attitude (“How much do you think the target behaviors are clever, interesting, nice, cool, and tasty?”) were used as described by Ajzen and Fishbein and as previously used in comparable research [16,27,31]. From these questions, a mean summary score for (1) attitude towards the FV product assessed in the questionnaire and (2) general attitude towards healthy products were calculated by adding the scores of the questions that were answered and dividing them by the amount of answered questions. For both attitude scores, the mean summary score could range from 1 (negative attitude) to 5 (positive attitude). The questionnaires were previously used in a pilot study regarding the learning street curriculum (not published) and appeared appropriate after small adaptations to the formulation of the questions.

### 2.5. Data Analysis

All analyses were performed using IBM SPSS Statistics for Windows (version 27.0, IBM Corp, Armonk, NY, USA). Only data from participants who completed at least 75% of the questionnaire were included in the present study. This percentage was based on comparable research, using the same cut-off point [27,32,33,34]. Baseline characteristics are presented as mean with standard deviation (SD) for numerical variables and as the number of participants with percentage (%) for categorical variables. The difference in these characteristics between the intervention and control group was investigated using independent-sample t-tests for the numerical variables and Pearson’s chi-square or Fisher’s exact tests, where appropriate, for the categorical variables. As for the main analyses, data were imputed for age, ethnicity, sex, and the different determinants of FV intake for each time point, using multiple imputations with 20 iterations and predictive mean matching. As the number of incomplete cases (at least one fixed variable missing and/or outcome variable missing at all time points) was 38%, at least 38 imputed datasets should be used according to the rule of thumb given by White et al. [35]. To be sure, 50 imputed datasets were created. A three-level linear mixed model analysis, with classes as the third level, participants as the second level, and measurements as the first level, was used to assess the longitudinal effect of the learning street on the various determinants assessed in the questionnaire. The fixed part of the model consisted of group (intervention versus control), time (T0, T1, T2), and the interaction term group * time to assess the group effect at each time point, correcting for the outcome at baseline. Furthermore, sex (male/female), age (in years, at T0), the FV product addressed in the learning street, and the baseline scores for the other four determinants of FV intake were included in the fixed part of the model. As for the random part of the model, a random intercept (and slope) on the class level was included next to an unstructured covariance structure for the three repeated measurements. We did not include the nesting of classes within schools as an additional level in our analysis as (a) the children mainly influence each other within a class and less within a school; (b) the number of included classes per school is very small; (c) the inclusion of schools as another level would further increase the number of levels in the analyses (there would be four levels), potentially resulting in estimation problems. As a sensitivity analysis, the same linear mixed model analyses were applied to the original (non-imputed) data. Furthermore, post-hoc analyses were performed, where we only corrected for the baseline outcome and those covariates and/or baseline scores of determinants of FV intake that were significantly different between the intervention and control group at baseline. Standardized effect sizes were calculated for each determinant and expressed as Cohen’s d, defined as the difference in observed mean change scores divided by the pooled standard deviation of the change scores. The Cohen’s d was interpreted as small (d = 0.2), medium (d = 0.5), and large (d = 0.8) [29]. Two-sided *p*-values ≤ 0.05 were considered statistically significant.

## 3. Results

### 3.1. Demographic Characteristics

Of the 91 children from the four classes (study years five and six) participating in a learning street program during the school year 2019–2020, 61 (67.0%) handed in a completed informed consent form so as to be included in the intervention group of the present study. Of these children, 60 met the additional inclusion criteria to be included in the present study’s data analyses. Of the 312 children from the 30 school classes already participating in the other research project, 165 children (52.8%) from 17 classes (study years five and six) were suitable for inclusion in the present study’s control group as they were from classes that were not (planning on) implementing nutrition-related and/or PA-related health-promoting initiatives in the context of the project. Of this group, 132 children from 15 classes were included as they met the additional criteria to be included in the present study’s data analyses. A detailed overview of the inclusion process of the participants can be found in Supplementary Figure S1.

Table 1 provides an overview of the sample’s baseline characteristics. The intervention and control group were comparable regarding sex and ethnicity. There were, however, significant baseline differences in age and intention to consume the FV item between the two groups. The mean age in the intervention group was significantly lower (8.3 years) compared to that of the control group (8.6 years) (*p* = 0.00). The baseline intention was significantly higher in the intervention group (3.9) compared to that of the control group (3.1) (*p* < 0.001).

### 3.2. Intervention Effects on the Psychosocial Determinants of FV Intake

**Figure 2:**
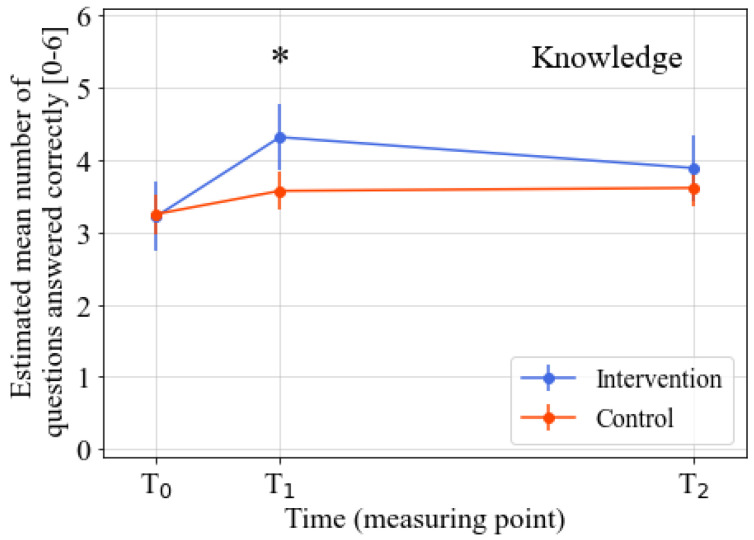
Estimated marginal means of the number of questions answered correctly from T0–T2. Note. Time span: T1–T0 = three weeks; T2–T0 = three months. * Significant difference between the intervention and control group (*p* ≤ 0.05). Analyzed by linear mixed model analyses. All analyses were corrected for baseline outcome, sex, age, FV product assessed in the questionnaire, and the baseline scores of the other four determinants of FV intake.

**Figure 3:**
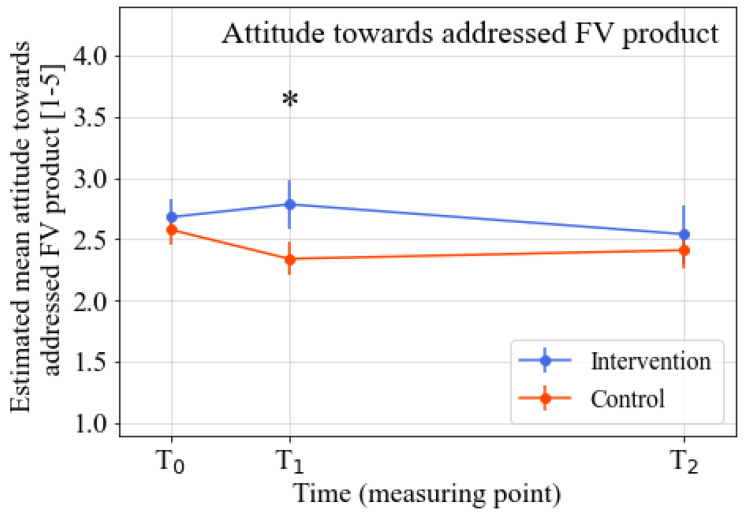
Estimated marginal means of taste preferences from T0–T2. Note. Time span: T1–T0 = three weeks; T2–T0 = three months. * Significant difference between the intervention and control group (*p* ≤ 0.05). Analyzed by linear mixed model analyses. All analyses were corrected for baseline outcome, sex, age, FV product assessed in the questionnaire, and the baseline scores of the other four determinants of FV intake.

**Figure 4:**
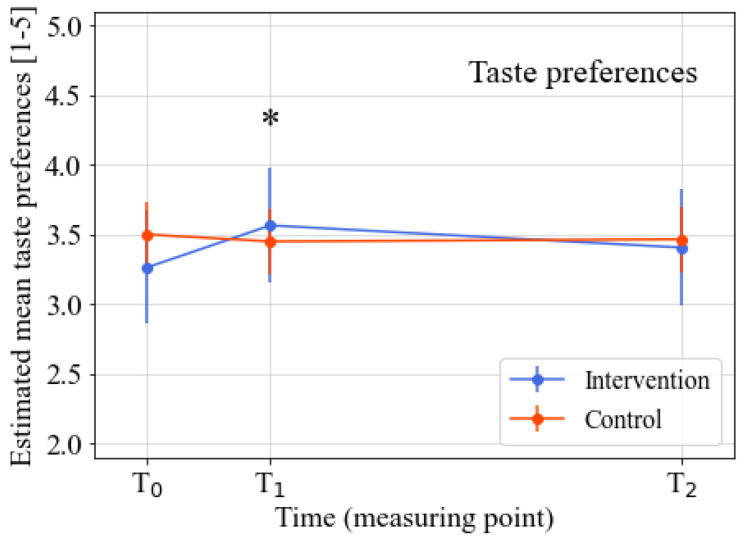
Estimated marginal means of attitude towards addressed FV product from T0–T2. Note. Time span: T1–T0 = three weeks; T2–T0 = three months. * Significant difference between the intervention and control group (*p* ≤ 0.05). Analyzed by linear mixed model analyses. All analyses were corrected for baseline outcome, sex, age, FV product assessed in the questionnaire, and the baseline scores of the other four determinants of FV intake.

**Figure 5:**
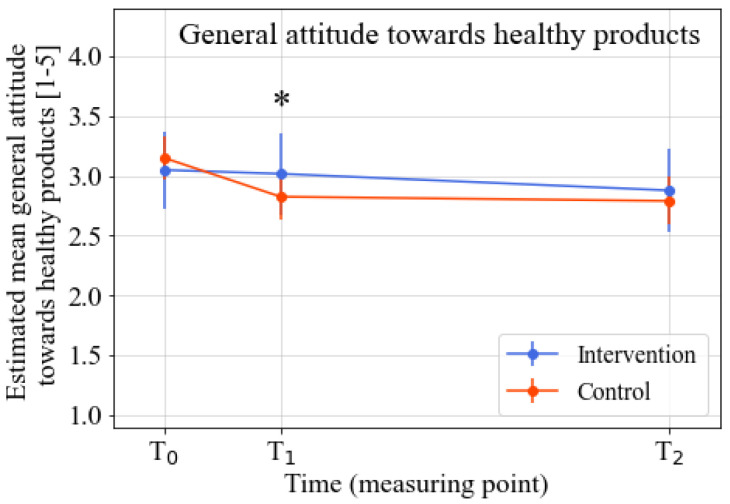
Estimated marginal means of general attitude towards healthy products from T0–T2. Note. Time span: T1–T0 = three weeks; T2–T0 = three months. * Significant difference between the intervention and control group (*p* ≤ 0.05). Analyzed by linear mixed model analyses. All analyses were corrected for baseline outcome, sex, age, FV product assessed in the questionnaire, and the baseline scores of the other four determinants of FV intake.

The intervention’s effects on the various psychosocial determinants were analyzed after correction for baseline knowledge, intention, taste preferences, attitude towards addressed FV product, general attitude towards healthy products, sex, age, and the FV product addressed in the learning street program. The analyses revealed significant positive intervention effects for the knowledge (*p* = 0.001; standardized effect size (ES = 0.60), taste preferences (*p* = 0.002; ES = 0.52), attitude towards addressed FV product (*p* = 0.004; ES = 0.48), and general attitude towards healthy products (*p* = 0.01; ES = 0.39) at T1 (directly after the intervention). No significant intervention effect for intention was found at T1. At T2 (three months after the intervention), the significant intervention effects for all outcomes had disappeared. Detailed information on the intervention effects for the various outcomes can be found in Table 2, Figure 2, Figure 3, Figure 4 and Figure 5, and Supplementary Figure S2. Descriptive data regarding the observed mean scores for the various determinants of FV intake from T0–T2 can be found in Supplementary Table S3. The estimated treatment effects based on the original (non-imputed) data can be found in Supplementary Table S4, showing similar effects. The post-hoc analyses, which were only corrected for the baseline outcome, age, and intention (as there were significant baseline differences between the intervention and control group for these two variables), also showed similar results (Supplementary Table S5).

## 4. Discussion

This study aimed to investigate the short-term and longer-term effects of participation in the Kokkerelli learning street on the knowledge, taste preferences, intention, attitude towards FV products, and general attitude towards healthy food products of Dutch primary school pupils aged 8–10 years old. For all determinants except intention, significant positive intervention effects were found over the short term (directly after the intervention). These intervention effects decreased over time and were no longer significant three months after the intervention. These findings are partly in line with our hypothesis, which described an expected increase over the short and long term for knowledge, taste preferences, intention, attitude towards addressed FV product, and general attitude towards healthy products following pupils’ participation in the learning street program.

The fact that no significant longer-term intervention effects were found for any of the study’s outcomes might be related to the intensity of the intervention. The learning street had a duration of approximately three weeks, during which children participated in various lessons and activities. However, after this period, schools continued with their regular curriculum. Additionally, all activities were organized within the school setting with limited efforts to include parents and the home setting in the learning street program. Besides the school environment, the home setting has a large influence on children’s dietary behaviors through factors such as FV availability at home and the dietary habits of family members [10,11,36]. The Dutch school-based intervention “Taste Lessons” was successful in achieving short-term and long-term improvements in children’s knowledge [16]. A possible explanation for the occurrence of long-term improvements in knowledge might be that Taste Lessons also stimulated parental involvement through homework assignments. Possibly, improvements in parental nutrition-related behaviors following the homework assignments positively influenced the children’s knowledge, as it is known that caregivers’ health-promoting behaviors are associated with higher nutrition-related knowledge and higher FV intake in children [37]. Another comparable intervention was the so-called “High 5 project”, although this intervention had a much higher intensity than the present intervention, including homework assignments, efforts to involve parents, and a food service component in the school cafeteria [38]. An evaluation of this intervention found long-term improvements in children’s knowledge and FV intake, although the magnitude of the effects had decreased at two-year follow-up compared with one-year follow-up [38]. The findings from these other interventions support the hypothesis that the present intervention’s intensity might not have been sufficient to achieve longer-term improvements in the various outcomes and that parental involvement might increase intervention impact. Indeed, a review by Contento et al. states that up to 50 classroom hours of exposure are needed to achieve stable improvements in various outcomes, which is considerably more than the (approximately) 4.5 h of exposure that was achieved in the present intervention. It also showed that programs with a duration of several years resulted in stable changes in dietary intake [39]. Repeating the present intervention several times a year and/or in various study years might therefore lead to more prominent effects. Despite the fact that Kids University for Cooking already recommends repeating the intervention over various study years, this is currently not a common practice due to various practical barriers mentioned by schools (e.g., limited space in the curriculum, limited financial resources, etc.).

The lack of observed intervention effects on intention could also potentially be linked to the intervention’s limited intensity and parental involvement. According to the theory of planned behavior, intention is the most important precedent for behavior. Intention is, in turn, influenced by attitude, subjective norms, and perceived behavioral control [40]. Potentially, the present intervention was not powerful enough to influence these determinants sufficiently to also lead to improvements in intention. It should also be noted that children’s FV intake and also, very likely, their intention to consume FV outside of school is largely regulated by parents and caregivers who purchase and prepare the food in the home setting. Possibly, children subconsciously took this large parental influence on their FV consumption behavior into account when answering the questions regarding their intention to consume FV. If no changes in parental behavior were expected, children might have reasoned that their own behavior and intention could not be changed either.

The evaluation of the effectiveness of Taste Lessons furthermore revealed a significant short-term increase in the number of different foods tasted by the intervention group, with the effect disappearing after six months [16]. These findings are in line with the significant short-term effects on taste preference which were observed in the present study [41,42]. Furthermore, the present study found a significant short-term improvement in attitude, while Taste Lessons was not successful in improving attitude over the short or long term. A possible explanation for this discrepancy in the findings might be the difference in the ratio between experiential learning and traditional learning in the two programs. Taste Lessons consisted of a large portion of traditional learning (in the form of classroom-based lessons combined with small experiments and activities), while the learning street program comprises more elements focusing almost exclusively on experiential learning (e.g., a visit to a grower’s farm and a cooking lesson) [43]. More experiential learning might lead to greater effects; research suggests it is a useful strategy to improve children’s attitudes [26]. Other studies evaluating interventions consisting of a large portion of experiential learning also found positive intervention effects on children’s attitudes over the short and/or long term [44,45,46].

### 4.1. Strengths and Limitations

The present study had several strengths and limitations. Various studies are available in which the effects of school-based nutrition education programs are investigated. However, the majority of these programs focus on traditional education or experiential learning strategies in isolation. To our knowledge, the present study is one of the few studies that has investigated the effects of a school-based nutrition program, deploying both traditional and experiential learning strategies [21], and has, therefore, contributed to the evidence base regarding this type of education program. Furthermore, not only the short-term effects immediately after the intervention were investigated but also the longer-term effects three months after the intervention. Although no significant longer-term effects were found, the study provides valuable insights and suggestions for improving interventions to increase their effectiveness (e.g., through increasing its intensity and including a parental component). In this way, this study provides valuable information that can help intervention developers, researchers, schools, and other stakeholders in the field to maximize the impact of future school-based nutrition education programs. A limitation of the study is the restricted external validity of the study’s results as the sample size is relatively small (especially in the intervention group), with a limited variety of ethnicity. It would, therefore, be beneficial to study the intervention’s effects in a larger and more diverse population. Furthermore, the potential occurrence of selection bias in the intervention group could not be investigated, as the researchers had no access to the demographic characteristics of children for whom no informed consent for study participation was obtained. A second limitation is the fact that no randomization took place when assigning schools to the intervention and control groups. This represented the real-world situation, as schools can enroll in the learning street program based on their willingness to participate. The fact that the control group consisted of classes already participating in another research project limited the external validity, although we made sure that these classes were not (planning on) implementing nutrition-related and/or PA-related health-promoting initiatives and were comparable with the classes from the intervention group. We tested for baseline differences between the intervention and control group and only found significant differences in age and intention. For age, it is debatable if this significant difference is relevant, as the mean difference between the intervention and control group was only approximately four months. For intention, the intervention group’s baseline mean was significantly higher than that of the control group. Although we corrected for this baseline difference in the analyses, this still might have influenced the results, as there might have been limited room for improvement due to the already high intention at baseline.

All analyses were corrected for baseline knowledge, intention, taste preferences, attitude towards addressed FV product, general attitude towards healthy products, sex, age, and the FV product addressed in the learning street. Furthermore, post-hoc analyses, where we only corrected for baseline outcome, age, and intention (as there were significant baseline differences between the intervention and control group for these two variables), were performed (Supplementary Table S4). As the results from the main analyses and the post-hoc analyses were comparable, it was determined that no overfitting had occurred and that the results from the main analyses were acceptable.

For various determinants, especially the determinants ‘intention’ and ‘taste preferences’, the number of missing values at all time points was relatively high. This was due to the answer option “I do not know”, which was often selected and was recoded as a missing value. Many participants selected this answer option because they were not familiar with the FV product prior to it being addressed in the learning street program. To compensate for the missing values, the data in the present study were imputed using multiple imputations.

Other important limitations are related to the measurement instrument that was used for the assessment of the various determinants of the children’s FV intake. The danger in using subjective measurements like questionnaires is that they might lead to social desirability bias [47]. However, there is currently no objective way to measure constructs such as taste preferences, intention, and attitude. During the administration of the questionnaire, the teachers tried to limit the occurrence of social desirability bias by stressing confidentiality and by telling participants there were no wrong or right answers. The used questionnaire was based on questionnaires used in comparable research [16], were tested in a pilot study on the learning street curriculum (not published) and appeared appropriate after making small adaptations to the formulation of the questions. However, it should be noted that the reliability and validity of the questionnaire were not specifically tested and are, therefore, not known. Furthermore, differences in reading and comprehension skills might have had an influence on the capacity of participants to answer the questionnaire adequately.

The fact that each learning street covered a different FV product and that participants from different classes, therefore, completed questionnaires concerning various products might have had an influence on the outcomes evaluated in the present study. Fruit items, like strawberries, are sweeter than vegetables, such as cauliflowers. Studies show that children prefer sweet products above bitter products [48,49], which makes it more difficult to compare the different FV products concerning outcomes such as taste preference. To avoid these differences playing a role in future evaluations, it would be better to investigate the effects of one specific learning street at a time (e.g., only evaluating the effects of the learning street program on cauliflower), which was not possible in the present study due to the relatively small sample size.

Another factor that had an impact on the present study was the outbreak of COVID-19 in early 2020. Due to this outbreak, Dutch schools were forced to close for several periods and could not participate in the learning street program during these periods. This meant that only four intervention classes could be included in the present study instead of the seven classes that were anticipated prior to the start of the study. As a response to this, it was decided to include as many control classes as possible to increase the statistical power. This resulted in the inclusion of 15 control classes, meaning that the control group (n = 132) was approximately twice as big as the intervention group (n = 60). Despite this large difference in the number of participants between the intervention and control group, including this many participants in the control group was still beneficial, as it has been shown that statistical power improves until the largest group is approximately three times the smallest group [50]. Furthermore, performing an effect size calculation with the same assumptions as described earlier, but with four intervention classes and 15 control classes, resulted in a Cohen’s d of 0.53 (was 0.52), indicating that the difference in the number of included classes did not have a large effect on the effect size. The COVID-19 outbreak and the subsequent school closures also meant that the timing of the questionnaire administration was different for the intervention and control groups. It is unknown what influence COVID-19 and the subsequent safety measures (e.g., school closures) had on the children’s (determinants of) FV intake, as studies report mixed effects of the pandemic on children’s FV consumption [51,52,53].

### 4.2. Implications for Research and Practice

Recommendations for future research include using a larger and/or more diverse population, exploring other measurement instruments to investigate children’s (determinants of) FV intake, evaluating the intervention’s effects on children’s FV intake, and investigating the intervention’s effects by evaluating a learning street which addresses one FV product at a time.

There are also various recommendations to further improve the learning street program itself. To achieve more sustainable intervention effects, the intensity of the learning street program should be increased. This can be achieved through a prolonged intervention duration, higher frequency of the provided lessons, and/or repeated participation in the learning street multiple times a year or for several school years in a row (as recommended by Kids University for Cooking). Furthermore, efforts to stimulate parental involvement could be a valuable addition to the learning street program. Parents play an important role in the development of children’s eating habits; therefore, it would be beneficial to target the home environment as well. This can be achieved by, e.g., expanding the learning street program to include homework assignments and/or family activities. The above-mentioned recommendations are not only valuable for the learning street intervention, but they are also relevant for the developers of other school-based nutrition education programs. Considering the potential impact of school-based nutrition education programs on children’s determinants of FV intake, we recommend including these programs as a mandatory part of the curriculum, something which is currently not the case in the Netherlands. This should, however, be carried out in close consultation with intervention developers and researchers to ensure that schools implement adequate and evidence-based programs. Furthermore, efforts should be made to reduce the various barriers that schools experience with regard to the implementation of nutrition education programs (e.g., limited space in the curriculum, high workload, and limited financial resources).

## 5. Conclusions

The present study showed the significant positive short-term effects of participating in the Kokkerelli learning street intervention on children’s knowledge, taste preferences, attitude towards the FV products addressed in the intervention, and their general attitude towards healthy food products. The results indicate that participation in the learning street program can contribute to behavioral change. However, the observed effects were not sustained over the long term. This might be explained by the relatively small study sample, issues related to the used measurement instrument, and/or the limited intensity and parental involvement in the learning street program. Longer-term effects might be achieved by repeating the program more often (e.g., multiple times a year and/or multiple school years in a row) and/or by including additional components in the learning street program (e.g., homework assignments to stimulate parental involvement). Further improving and investigating the learning street program and comparable interventions is highly relevant to gain more insight into ways to sustainably improve children’s determinants of FV intake and, subsequently, their overall health.

## Figures and Tables

**Table 1:** Baseline characteristics of participants from the present study’s intervention and control group (n = 190).

	Intervention Group				Control Group					
	n	% Missing Values ^1^	%/M	SD	n	% Missing Values ^1^	%/M	SD	Chi^2^/*t*-Value	*p*-Value
Schools	4	n/a	n/a	n/a	7	n/a	n/a	n/a	n/a	n/a
Included classes per school	1	n/a	n/a	n/a	2–4 ^2^	n/a	n/a	n/a	n/a	n/a
Included participants per class	n/a	n/a	15.0	6.5	n/a	n/a	8.8	3.8	n/a	n/a
Sex (% boys)	24	3.3	41.4		55	0.0	41.7		0.00	0.97
Age (years)	60	0.0	8.3	0.5	132	0.0	8.6	0.7	−3.81	<0.001 *
Ethnicity (%Western) ^3^	52	6.7	92.9		127	0.8	97.0			0.24
Knowledge (mean correct) [0–6]	60	0.0	3.0	1.6	132	0.0	3.2	1.2	−0.75	0.46
Intention (mean score) [1–5]	46	23.3	3.9	1.1	96	27.3	3.1	1.3	3.50	<0.001 *
Taste preferences (mean score) [1–5]	54	10.0	3.6	1.1	115	12.9	3.5	1.2	0.44	0.66
Attitude towards addressed FV product (mean score) [1–5]	59	1.7	2.7	0.9	131	0.8	2.6	0.9	0.81	0.42
General attitude towards healthy food (mean score) [1–5]	58	3.3	3.0	0.9	131	0.8	3.1	0.9	−1.01	0.31

Note. All children who filled in 75% of the questionnaire and 67% of the questions for each determinant at T0 were included in the baseline. Children who were not present at T0 were not included. For intention, a five-point Likert scale was used: 1: “No, I do not want to”, 2: “I do not think so”, 3: “Maybe”, 4: “I think so”, 5: “Yes I want to”. For taste preferences, a comparable scale was used: 1: “I do not like it”, 2: “I do not really like it”, 3: “It is okay”, 4: “I like it”, 5: “I like it very much”. For attitude, the following scale was used: 1: “No, sure not”, 2: “I do not think so”, 3: “In between”, 4: “Yes, I think so”, 5: “Yes, sure”. Abbreviations; FV: fruit and vegetables, n: number of participants, M: mean, SD: standard deviation. * Significant difference between intervention and control group (*p* ≤ 0.05); ^1^ missing values based on true missings and participants having selected the answer option “I do not know”; ^2^ in five schools, two classes were included. In one school, three classes were included. In one school, four classes were included; ^3^ Fisher’s exact test.

**Table 2:** Estimated Treatment Effects after Multiple Imputation (n = 192).

Determinant		Intervention vs. Control		
		B (95% CI)	*p*-Value	ES ^1^
Knowledge	T1−T0	0.78 (0.32; 1.23)	0.001 *	0.60
	T2−T0	0.31 (−0.28; 0.90)	0.31	0.22
Intention	T1−T0	0.11 (–0.24; 0.45)	0.55	0.20
	T2−T0	–0.21 (–0.65; 0.22)	0.33	–0.09
Taste preferences	T1−T0	0.35 (0.13; 0.57)	0.002 *	0.52
	T2−T0	0.18 (–0.07; 0.42)	0.16	0.20
Attitude towards addressed FV product	T1−T0	0.32 (0.10; 0.55)	0.004 *	0.48
	T2−T0	0.03 (–0.25; 0.30)	0.84	0.00
General attitude towards healthy products	T1−T0	0.29 (0.06; 0.53)	0.01 *	0.39
	T2−T0	0.19 (–0.09; 0.47)	0.19	0.19

Note. Time span: T1–T0 = three weeks; T2–T0 = three months. Abbreviations; CI: confidence interval, ES: effect size, FV: fruit and vegetables. * Significant difference between the intervention and control group (*p* ≤ 0.05), analyzed by linear mixed model analyses. All analyses were corrected for baseline outcome, sex, age, FV product assessed in the questionnaire, and the baseline scores of the other four determinants of FV intake. ^1^ Effect size based on observed means and SD from original data, not imputed data.

## Data Availability

Data will be available upon request. Data will only be shared with parties who provide a methodologically-sound proposal, or for individual participant data meta-analysis. Proposals should be directed to mth.hahnraths@maastrichtuniversity.nl. To gain access, data requestors will need to sign a data access agreement.

## Supplementary Materials

The following supporting information can be downloaded at: https://www.mdpi.com/article/10.3390/nu14204259/s1, Supplementary Table S1: Overview of the Various Components of Each Learning Street; Supplementary Table S2: Overview of the Theoretical Foundations of the Learning Street; Supplementary Table S3: Observed Mean Scores for the Various Determinants of FV Intake at the Various Time Points (T0–T2); Supplementary Table S4: Estimated Treatment Effects Based on Original (Non-Imputed) Data; Supplementary Table S5: Post-hoc analyses–Estimated Treatment Effects after Multiple Imputation and Corrected for Baseline Outcome, Age, and Intention Assessed in the Questionnaire. Figure S1: Flowchart Study Participation; Supplementary Figure S2: Estimated Marginal Means for Intention at T0–T2.
