# Supplementary material for: The Effects of a Multi-Component School-Based Nutrition Education Intervention on Children’s Determinants of Fruit and Vegetable Intake
Source: Nutrients. 2022 Oct 12;14(20):4259. doi: 10.3390/nu14204259 (PMC9607228; doi:10.3390/nu14204259)
Supplement: Supplementary file 1 [file nutrients-14-04259-s001.zip › Figure S1. Flowchart Study Participation..pdf]

Figure S1. Flowchart Study Participation.

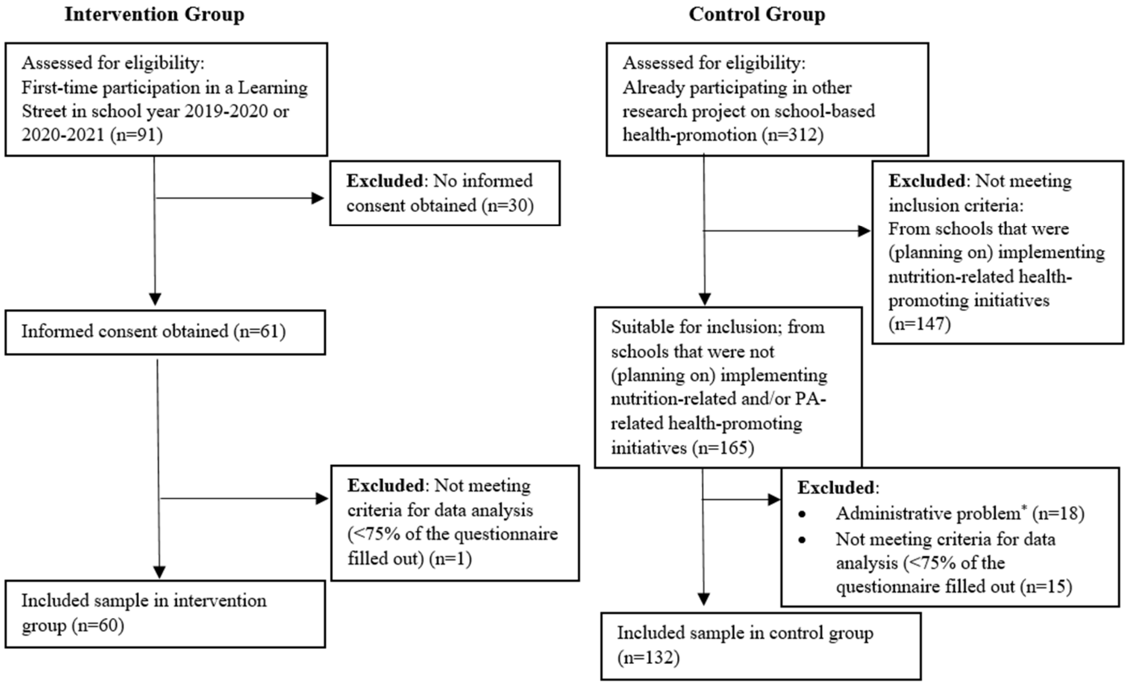

Figure S1. Flowchart Study Participation.

* Due to an administrative problem, the questionnaire for the wrong FV product was filled out by 18 participants at T1, which resulted in their exclusion from the study.
