# Supplementary material for: The Effects of a Multi-Component School-Based Nutrition Education Intervention on Children’s Determinants of Fruit and Vegetable Intake
Source: Nutrients. 2022 Oct 12;14(20):4259. doi: 10.3390/nu14204259 (PMC9607228; doi:10.3390/nu14204259)
Supplement: Supplementary file 1 [file nutrients-14-04259-s001.zip › Figure S2. Intention at T0-T2..pdf]

Figure S2. Estimated Marginal Means for Intention at T0-T2.

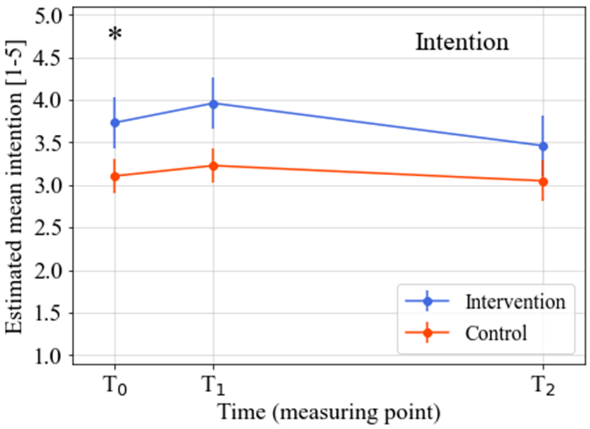

Figure S2. Estimated Marginal Means for Intention at T0-T2.
Note. Time span: T1-T0=three weeks; T2-T0=three months.
*Significant difference between intervention and control group ($p \leq 0.05$).
Analysed by linear mixed model analyses. All analyses were corrected for baseline outcome, sex, age, FV product assessed in the questionnaire, and the baseline scores of the other four determinants of FV intake.
