# Supplementary material for: The Effects of a Multi-Component School-Based Nutrition Education Intervention on Children’s Determinants of Fruit and Vegetable Intake
Source: Nutrients. 2022 Oct 12;14(20):4259. doi: 10.3390/nu14204259 (PMC9607228; doi:10.3390/nu14204259)
Supplement: Supplementary file 1 [file nutrients-14-04259-s001.zip › Table S1. Overview of the Learning Street..pdf]

Table S1. Overview of the Various Components of Each Learning Street.

Table S1. Overview of the Various Components of Each Learning Street.

Intervention Component	Description	Duration and Location	Materials	Delivery Channel
**1. Introduction lesson**	During the introduction lesson, children are introduced to (the taste of) the product. Children are familiarised with growing and harvesting processes, as well as with the importance of the product regarding health.	45 minutes at school	• Preparation of the product (e.g., three types of mushrooms). • Poster of the food guide pyramid. • Poster with information on unnecessary food wastage. • PowerPoint presentation for assistance during the lesson.	Delivered by the responsible teacher, with help of guidelines and materials provided by Kids University for Cooking Foundation.
**2. Visit to the grower's farm**	During the visit to the grower's farm, children are introduced to the precise planting, growing and harvesting procedures of the product. Children are allowed to enter the facilities (e.g., the greenhouse) and to closely observe and experience the farming of the product.	60 minutes at the grower's farm	No necessary material	Delivered by the farmer in cooperation with Kids University for Cooking Foundation.
**3. Cooking**	Children observe and listen to a professional chef, while he/she explains each step that has to be taken for the preparation of the meal. Subsequently, children prepare their own portion of the meal with help of volunteers. After preparing the meal, children help to set the table and consume their self-prepared meal together.	60-90 minutes at the cooking facilities of Kids University for Cooking	• Cooking facilities such as a kitchen. • Cutting boards and knifes suitable for children. • Ingredients for the pre-chosen meals.	Delivered by a professional chef, assisted by volunteers.
**4. Evaluation lesson**	Children evaluate the learning street together and discuss what they have learned.	45 minutes at school	No necessary material.	Delivered by the responsible teacher, with help of guidelines provided by Kids University for Cooking Foundation.
