# Supplementary material for: The Effects of a Multi-Component School-Based Nutrition Education Intervention on Children’s Determinants of Fruit and Vegetable Intake
Source: Nutrients. 2022 Oct 12;14(20):4259. doi: 10.3390/nu14204259 (PMC9607228; doi:10.3390/nu14204259)
Supplement: Supplementary file 1 [file nutrients-14-04259-s001.zip › Table S2. Theoretical Foundations..pdf]

Table S2. Overview of the Theoretical Foundations of the Learning Street.

Table S2. Overview of the Theoretical Foundations of the Learning Street.

Determinant	Theoretical method	Practical application/Hypothesised effects	Intervention components
Attitude	Participation	High levels of engagement during all group activities lead to larger impact on the individual.	1. Introduction lesson<br>2. Visit at the grower's farm<br>3. Cooking<br>4. Evaluation lesson
	Modelling	Close observation of an appropriate model performing health related behaviour stimulates individuals' response.	2. Visit at the grower's farm<br>3. Cooking
	Environmental re-evaluation	Encouraging the positive effects of the healthy food within the individual's environment leads to larger impact on the individual.	2. Visit at the grower's farm<br>3. Cooking
	Direct experience	Direct contact with FV increases positive attitudes; it offers the possibility to try new behaviours first-hand.	1. Introduction lesson<br>2. Visit at the grower's farm<br>3. Cooking
Subjective norms	Participation	High levels of engagement during all group activities lead to larger impact on the individual.	2. Visit at the grower's farm<br>3. Cooking<br>4. Evaluation lesson
	Providing opportunity for social support	The surrounding of peers experiencing a stimulating learning environment stimulates the feeling of unity.	3. Cooking<br>4. Evaluation lesson
Self-efficacy	Active learning	First-hand experience in a stimulating environment enhances learning processes and confidence in one's own ability.	2. Visit at the grower's farm<br>3. Cooking
	Tailoring	Environment and intervention components suitable for children increase children's interest and abilities.	1. Introduction lesson<br>2. Visit at the grower's farm<br>3. Cooking
	Modelling	Close observation of an appropriate model performing health-related behaviour stimulates individuals' response.	2. Visit at the grower's farm<br>3. Cooking

Table S2. Overview of the Theoretical Foundations of the Learning Street *(cont.)*.

Determinant	Theoretical method	Practical application/Hypothesised effects	Intervention components
Self-efficacy	Guided practice	Precise guidance of an expert increases children's beliefs in their own abilities.	1. Introduction lesson<br>2. Visit at the grower's farm<br>3. Cooking
	Enactive mastery experience	Positive outcomes of newly learned behaviours enhance the beliefs in one's own ability.	3. Cooking<br>4. Evaluation lesson
Knowledge	Active learning	First-hand experience in a stimulating environment enhances learning processes.	2. Visit at the grower's farm<br>3. Cooking
	Tailoring	Environment and intervention components suitable for children increase children's knowledge.	2. Visit at the grower's farm<br>3. Cooking<br>4. Evaluation lesson
	Discussion	Open discussion about health-related topics increases knowledge in an interactive manner.	1. Introduction lesson<br>3. Cooking<br>4. Evaluation lesson
	Direct experience	Direct contact with FV offers better learning experience.	1. Introduction lesson<br>2. Visit at the grower's farm<br>3. Cooking
	Guided practice	Precise guidance of an expert increases children's knowledge and skills.	3. Cooking
Intention	Active learning	First-hand experience in a stimulating environment enhances intentional beliefs.	2. Visit at the grower's farm<br>3. Cooking
	Environmental re-evaluation	Encouraging the positive effects of the healthy food within the individual's environment leads to larger impact on the individual.	2. Visit at the grower's farm<br>3. Cooking
	Direct experience	Direct contact with FV leads to higher intentional beliefs.	1. Introduction lesson<br>3. Cooking
Taste preferences	Active learning	First-hand experience in a stimulating environment enhances interest in new tastes.	1. Introduction lesson<br>3. Cooking

Table S2. Overview of the Theoretical Foundations of the Learning Street *(cont.)*.

Determinant	Theoretical method	Practical application/Hypothesised effects	Intervention components

Taste preferences	Facilitation	More encounters with higher FV variety lead to higher taste preference.	3. Cooking
Habitual eating patterns	Counter-conditioning	Providing healthier choices to normal behaviours and reinforcing the healthy choices leads to the development of new habits.	3. Cooking
Motivational regulation	Participation	High levels of engagement during all group activities lead to larger impact on the individual.	2. Visit at the grower's farm<br>3. Cooking
	Active learning	First-hand experience in a stimulating environment enhances motivation among children.	2. Visit at the grower's farm<br>3 Cooking
	Tailoring	Offering a child-friendly learning environment enhances motivation among participants.	2. Visit at the grower's farm<br>3. Cooking
	Modelling	Close observation of an appropriate model performing health-related behaviour stimulates individuals' response.	2. Visit at the grower's farm<br>3. Cooking

Abbreviations: FV, fruit and vegetables.
