# Supplementary material for: The Effects of a Multi-Component School-Based Nutrition Education Intervention on Children’s Determinants of Fruit and Vegetable Intake
Source: Nutrients. 2022 Oct 12;14(20):4259. doi: 10.3390/nu14204259 (PMC9607228; doi:10.3390/nu14204259)
Supplement: Supplementary file 1 [file nutrients-14-04259-s001.zip › Table S3. Observed mean scores at T0-T2..pdf]

Table S3. Observed mean scores for the various determinants of FV intake at the various time points (T0-T2).

Table S3. Observed mean scores for the various determinants of FV intake at the various time points (T0-T2).

Determinant	T0				T1				T2			
	Intervention group		Control group		Intervention group		Control group		Intervention group		Control group	
	n	*Mean (SD)*	*n*	*M (SD)*	*n*	*M (SD)*	*n*	*M (SD)*	*n*	*M (SD)*	*n*	*M (SD)*
Knowledge (mean correct) [0-6]	60	3.0 (1.58)	132	3.2 (1.21)	57	4.1 (1.09)	128	3.5 (1.33)	53	3.7 (1.31)	131	3.6 (1.24)
Intention (mean score) [1-5]	46	3.9 (1.08)	96	3.1 (1.27)	48	4.1 (0.91)	95	3.2 (1.25)	41	3.8 (1.28)	91	3.3 (1.44)
Taste preferences (mean score) [1-5]	54	3.6 (1.10)	115	3.5 (1.19)	55	3.8 (1.08)	119	3.4 (1.18)	52	3.7 (1.06)	120	3.4 (1.21)
Attitude towards addressed FV product (mean score) [1-5]	59	2.7 (0.93)	131	2.6 (0.90)	57	2.8 (0.97)	128	2.3 (0.89)	54	2.6 (0.96)	132	2.4 (0.93)
General attitude towards healthy products (mean score) [1-5]	58	3.0 (0.91)	131	3.1 (0.89)	56	3.0 (0.93)	128	2.8 (0.97)	53	2.9 (1.05)	131	2.8 (0.89)

Note. All children who filled in 75% of the questionnaire and 67% of the questions for each determinant at the different time points were included in the sample.
For intention, a five-point Likert scale was used; 1: "No, I do not want to", 2: "I do not think so", 3: "Maybe", 4: "I think so", 5: "Yes I want to". For taste preferences, a comparable scale was used; 1: "I do not like it", 2: "I do not really like it", 3: "It is okay", 4: "I like it", 5: "I like it very much". For attitude, the following scale was used; 1: "No, sure not", 2: "I do not think so", 3: "In between", 4: "Yes, I think so", 5: "Yes, sure".
Abbreviations; n: number of participants, M: mean, SD: standard deviation, FV: fruit and vegetables.
