# Supplementary material for: The Effects of a Multi-Component School-Based Nutrition Education Intervention on Children’s Determinants of Fruit and Vegetable Intake
Source: Nutrients. 2022 Oct 12;14(20):4259. doi: 10.3390/nu14204259 (PMC9607228; doi:10.3390/nu14204259)
Supplement: Supplementary file 1 [file nutrients-14-04259-s001.zip › Table S4. Effects Based on Original Data..pdf]

Table S4. Estimated Treatment Effects Based on Original (Non-Imputed) Data.

Table S4. Estimated Treatment Effects Based on Original (Non-Imputed) Data.

		**Intervention vs. control**	
**Determinant**		*B (95% CI)*	*p-value*
Knowledge (n=119)	T1-T0	0.99 (0.41;1.56)	0.001*
	T2-T0	0.50 (-0.26; 1.26)	0.18
Intention (n=154)	T1-T0	0.19 (-0.16; 0.53)	0.28
	T2-T0	-0.08 (-0.46; 0.29)	0.66
Taste preferences (n=130)	T1-T0	0.38 (0.14; 0.62)	0.002*
	T2-T0	0.22 (-0.04; 0.48)	0.10
Attitude towards addressed FV product (n=122)	T1-T0	0.31 (0.06; 0.56)	0.02*
	T2-T0	-0.01 (-0.43; 0.29)	0.70
General attitude towards healthy food (n=123)	T1-T0	0.28 (0.02; 0.54)	0.04*
	T2-T0	0.20 (-0.15; 0.54)	0.26

Note. Time span: T1-T0=three weeks; T2-T0=three months.
Abbreviations; ES: Effect Size, FV: fruit and vegetables.
*Significant difference between intervention and control group (p≤0.05).
Analysed by linear mixed model analyses. All analyses were corrected for baseline outcome, sex, age, FV product assessed in the questionnaire, and the baseline scores of the other four determinants of FV intake.
