# Supplementary material for: The Effects of a Multi-Component School-Based Nutrition Education Intervention on Children’s Determinants of Fruit and Vegetable Intake
Source: Nutrients. 2022 Oct 12;14(20):4259. doi: 10.3390/nu14204259 (PMC9607228; doi:10.3390/nu14204259)
Supplement: Supplementary file 1 [file nutrients-14-04259-s001.zip › Table S5. Post-hoc analyses..pdf]

Table S5. Post-hoc analyses - Estimated Treatment Effects after Multiple Imputation.

Table S5. Estimated Treatment Effects after Multiple Imputation (n=192).

		**Intervention vs. control**	
**Determinant**		*B (95% CI)*	*p-value*
Knowledge	T1-T0	0.77 (0.32; 1.23)	0.001*
	T2-T0	0.30 (-0.29; 0.89)	0.32
Intention	T1-T0	0.11 (-0.24; 0.46)	0.53
	T2-T0	-0.14 (-0.67; 0.39)	0.60
Taste preferences	T1-T0	0.35 (0.13; 0.57)	0.002*
	T2-T0	0.18 (-0.07; 0.42)	0.16
Attitude towards addressed FV product	T1-T0	0.32 (0.10; 0.55)	0.004*
	T2-T0	0.03 (-0.25; 0.30)	0.84
General attitude towards healthy food	T1-T0	0.29 (0.06; 0.53)	0.01*
	T2-T0	0.19 (-0.09; 0.47)	0.19

Note. Time span: T1-T0=three weeks; T2-T0=three months.
Abbreviations; ES: Effect Size, FV: fruit and vegetables.
*Significant difference between intervention and control group ($p \leq 0.05$).
Analysed by linear mixed model analyses. All analyses were corrected for baseline outcome, age, and baseline intention.
